# The protein kinase CK2 contributes to the malignant phenotype of cholangiocarcinoma cells

**DOI:** 10.1038/s41389-019-0171-x

**Published:** 2019-10-22

**Authors:** Giovanni Di Maira, Alessandra Gentilini, Mirella Pastore, Alessandra Caligiuri, Benedetta Piombanti, Chiara Raggi, Elisabetta Rovida, Monika Lewinska, Jesper B. Andersen, Christian Borgo, Mauro Salvi, Daniele Ottaviani, Maria Ruzzene, Fabio Marra

**Affiliations:** 1 Dipartimento di Medicina Sperimentale e Clinica and Center Denothe, Università di Firenze, Florence, Italy; 2 Dipartimento di Scienze Biomediche Sperimentali e Cliniche, Università di Firenze, Florence, Italy; 3 Biotech Research and Innovation Centre (BRIC), Department of Health and Medical Sciences, University of Copenhagen, Copenhagen, Denmark; 4 Dipartimento di Scienze Biomediche, Università di Padova, Padua, Italy; 5 Present Address: UCL Institute of Ophthalmology, 11-43 Bath Street, London, EC1V 9EL UK

**Keywords:** Apoptosis, Biliary tract cancer

## Abstract

Cholangiocarcinoma (CCA) is a particularly aggressive hepatobiliary malignancy, for which the molecular mechanisms underlying the malignant phenotype are still poorly understood, and novel and effective therapeutic strategies are limited. The pro-survival protein kinase CK2 is frequently overexpressed in cancer and is receiving increasing interest as an anti-tumor drug target. Its precise role in CCA biology is still largely unknown. Here we show that expression of the CK2α and α’ catalytic subunits and of the β regulatory subunit is increased in human CCA samples. Increased expression of CK2 subunits was shown in CCA cell lines compared to non-transformed cholangiocytes. We used chemical inhibition of CK2 and genetic modification by CRISPR/Cas9 to explore the contribution of CK2 to the malignant phenotype of CCA cells. Disruption of CK2 activity results in cell death through apoptosis, reduced invasion and migration potential, and G0/G1 cell cycle arrest. Importantly, CCA cells with a reduced CK2 activity are more sensitive to chemotherapy. Altogether, our results demonstrate that CK2 significantly contributes to increased proliferative potential and augmented growth of CCA cells and indicate the rationale for its targeting as a promising pharmacologic strategy for cholangiocarcinoma.

## Introduction

Cholangiocarcinoma (CCA) is a lethal form of cancer which accounts for approximately 10–25% of all hepatobiliary malignancies^1^. Diagnosis in early stage of disease is very difficult due to lack of specific symptoms and biomarkers and therefore, the prognosis is very poor^2^. Like most cancers, development of CCA is a multistep process characterized by genetic and epigenetic alterations in pivotal genes leading to the activation of oncogenes and inactivation or loss of tumor suppressor genes^3,4^. Given the aggressive nature of CCA and the absence of effective therapeutic options, there is an unmet need for identification of novel therapeutic targets. However, despite significant advancement in the understanding of CCA pathogenesis, little is known about the molecular mechanism underlying the pro-tumorigenic properties of CCA cells.

CK2 is a constitutively active serine/threonine protein kinase found in cells as a tetrameric enzyme, and is composed of two catalytic subunits (α or its isoform α′) and two regulatory subunits (β), the latter controlling substrate-specificity and enzyme stability^5^. CK2 phosphorylates hundreds of substrates and is involved in many cellular processes^6^. While it is ubiquitously expressed, higher levels have been found in cancer cells as compared to healthy cells^7,8^. Despite being ubiquitous, CK2 is dispensable for normal cells^9^, while cancer cells rely on its activity for their growth^10^. Indeed, CK2 inhibition was proposed as an anticancer strategy^11–15^. Several CK2 inhibitors with cytotoxic properties in tumor cells and anti-cancer effects in animal models have been developed^16,17^. The CK2 inhibitor, CX4945^18^ has entered clinical trials in phase I/II for different types of cancer, including CCA where it is administered in combination with cisplatin plus gemcitabine (NCT02128282). Recently, the direct involvement of CK2 in CCA has been reported in a study showing that CX4945 has an anti-proliferative effect on CCA cells in vitro, and in mouse xenograft models^19^. Moreover, a study in primary CCA cells demonstrated a pro-apoptotic effect of CX4945 in correlation with CK2 expression levels, and suggested the potential benefit of a combined CK2/TGF-β targeting^20^. Here we provide a novel insight into the molecular mechanisms by which CK2 contributes to the malignant phenotype of CCA, exploiting pharmacologic and genetic approaches.

## Results

### CK2 status in human CCA and CCA cell lines

**Fig. 1:**
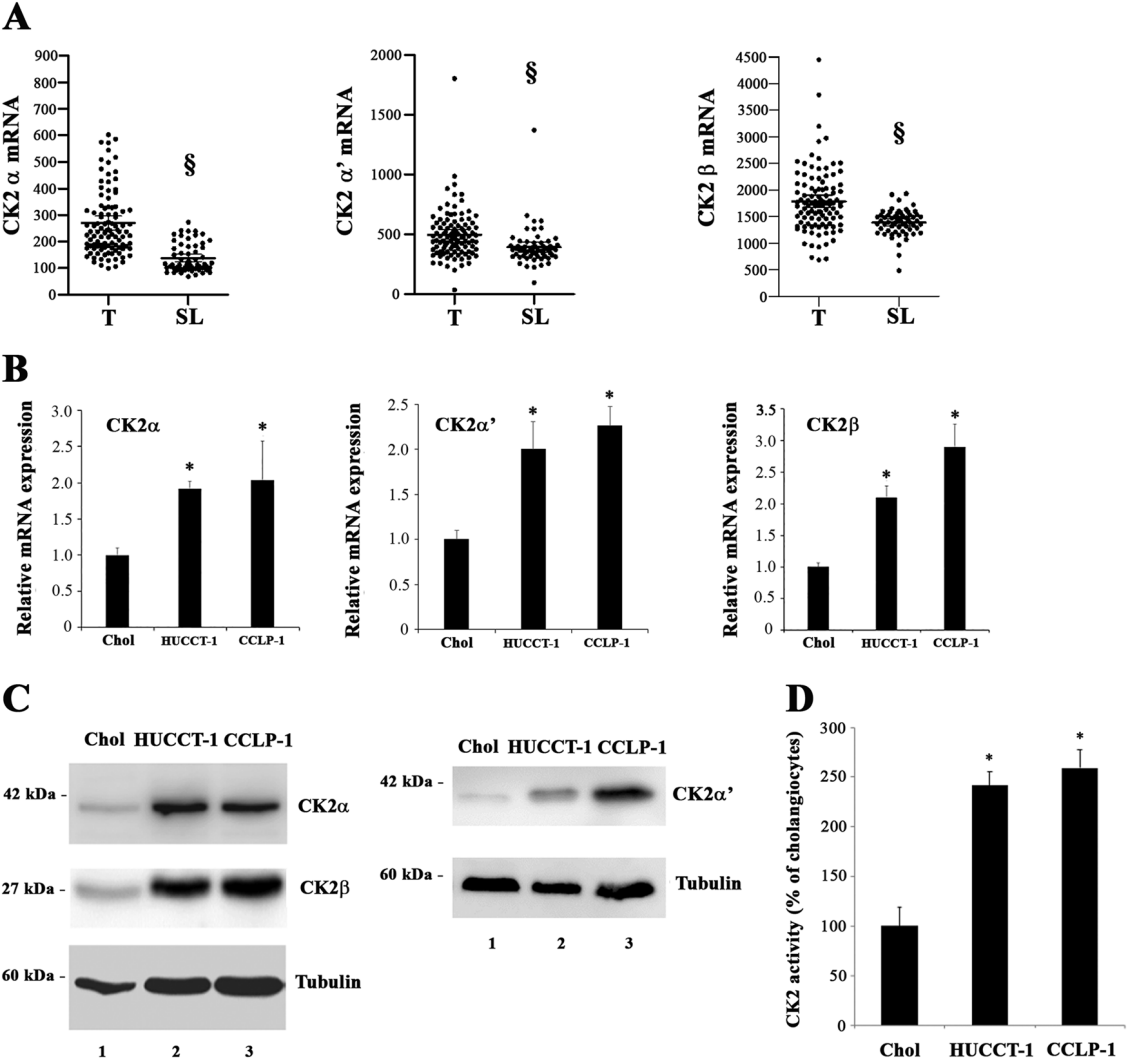
Expression and activity of CK2 in cholangiocarcinoma samples and CCA cells lines. **a** Expression of CK2 catalytic (CSNK2A1 and CSNK2A2) and regulatory (CSNK2B) subunits in human CCA tissues. CK2α, CK2α’ and CK2β mRNA expression were significantly increased in tumor lesions (T) (*n* = 104) compared to surrounding matched normal liver tissue (SL) (*n* = 60) using transcriptome data of CCA patients^35^. Statistical significance was determined by nonparametric Mann–Whitney test. Individual patient data are shown, and the thick horizontal line indicates the mean of the values. §, *P* < 0.001 vs. *T*. **b** Relative quantitation of gene expression of CK2α, CK2α’ and CK2β in cholangiocytes, HUCCT-1 and CCLP-1 cells. *p < 0.05 versus cholangiocytes. (**c**) Twenty µg (left panel) or 40 µg (right panel) of proteins from total cell lysate were analyzed by Western blot with the indicated antibodies. Tubulin was used as a loading control. **d** Activity of CK2 in cell lysates was measured using a CK2-specific peptide substrate. *p < 0.05 versus cholangiocytes

To establish whether CK2 expression is higher in CCA, we first analyzed transcriptomic data from 104 surgically resected CCA samples and matched surrounding livers. Transcripts of CK2α, CK2α′ and CK2β subunits were significantly increased in tumor samples compared to paired non*-*tumor liver tissue (Fig. 1a). Next, we examined the expression of CK2 subunits in two CCA cell lines, HUCCT-1 and CCLP-1 in comparison with non-transformed cholangiocytes. Significant overexpression of CK2α, CK2α′ and β subunits was evident in CCA cells compared to control cells, both at mRNA and protein levels (Fig. 1b, c). To assess if CK2 overexpression correlates with enhanced catalytic activity, we performed an in vitro kinase assay of whole cell extracts using a CK2-specific peptide as substrate (Fig. 1d). Consistently, CK2 activity was considerably higher in CCA cells than in non*-*neoplastic biliary epithelial cells. Altogether, these findings indicate that CK2 is elevated in CCA tissue and cells, thus suggesting a potential role for CK2 in the biology of CCA.

### Effect of the CK2 inhibitor CX4945 on CK2 activity and cell viability in CCA cells

**Fig. 2:**
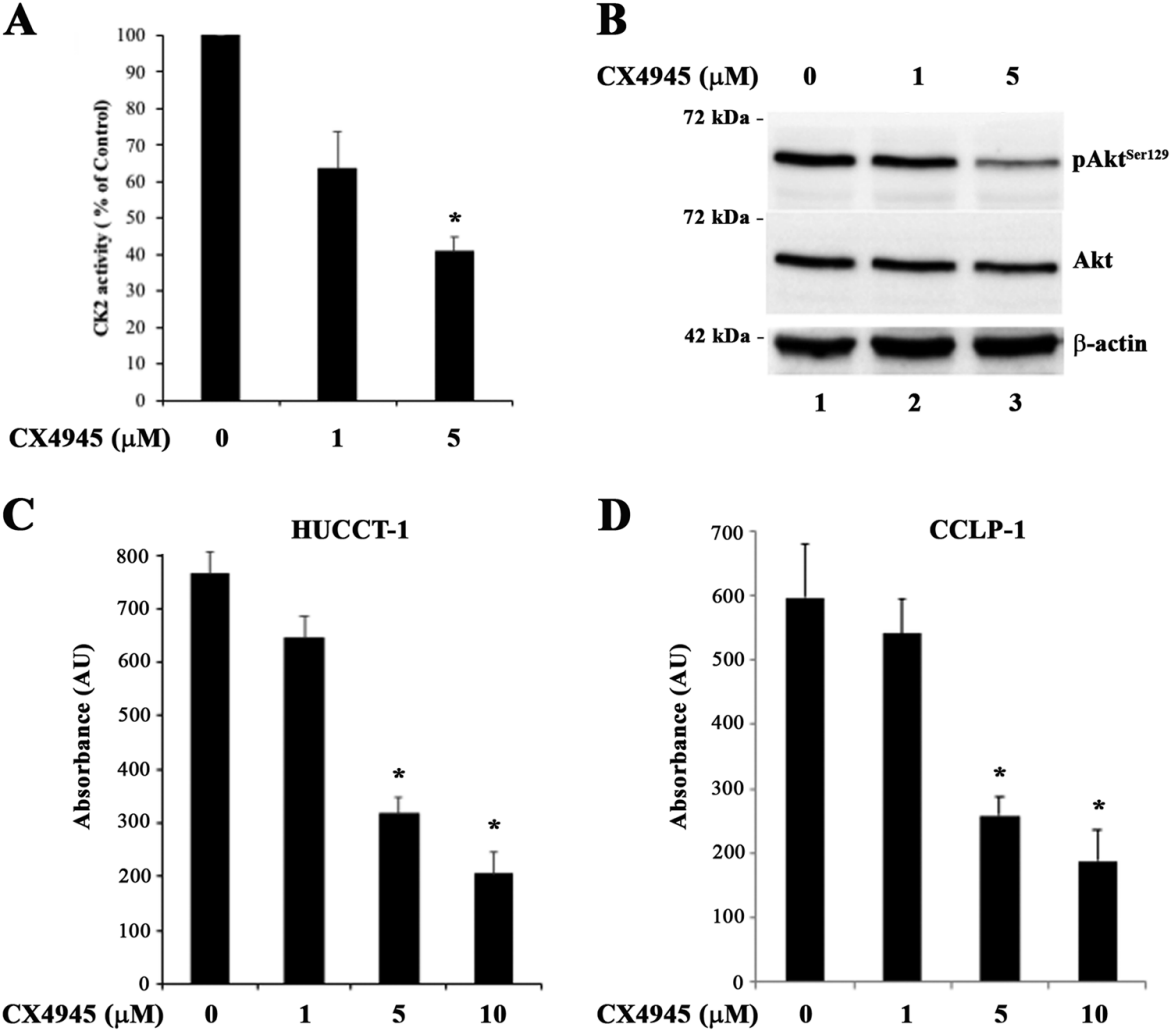
Effects of the CK2 inhibitor CX4945 on CK2 activity and cell viability in CCA cells. **a** Cultured HUCCT-1 cells were treated with vehicle or the indicated concentrations of CX4945 for 6 h. Activity of CK2 in cell lysates was measured towards the CK2-specific peptide substrate. *p < 0.05 versus vehicle. **b** Cultured HUCCT-1 cells were treated with vehicle or the indicated concentrations of CX4945 for 6 h. 25 µg of proteins from total cell lysate were analyzed by western blot with the indicated antibodies. **c**, **d** CCA cells were treated with the indicated concentrations of CX4945 for 48 h. MTT assay was performed at end of the treatment. *p < 0.05 versus control

To explore the role of CK2 in CCA cell biology, we treated cells with the CK2 inhibitor CX4945. To establish whether CX4945 effectively blocks CK2 activity in CCA cells, we measured CK2 enzymatic activity towards a specific peptide substrate and evaluated the phosphorylation state of the Akt^S129^, a CK2 intracellular substrate^21^. CK2 activity was reduced in a dose-dependent manner by CX4945 in HUCCT-1 cells (Fig. 2a), and the drop of CK2 kinase activity by CX4945 was confirmed by dephosphorylation of Akt^S129^ (Fig. 2b). Similar results were observed in CCLP-1 cells (data not shown).

Next, we assessed the effect of CK2 inhibition on CCA cell viability by means of a MTT assay. CCA cells cultured in the presence of increasing concentrations of CX4945 showed a dose-dependent reduction in cell viability (Fig. 2c, d). These results were confirmed by treating cells with 4,5,6,7-Tetrabromobenzotriazole (TBB), a CK2 inhibitor structurally unrelated to CX4945^22^ (Supplementary Fig. 1), thus confirming the relevant role of CK2 in this context, even considering possible off-target effects of CX4945^23,24^.

### CK2 inhibition induces apoptosis in CCA cells

**Fig. 3:**
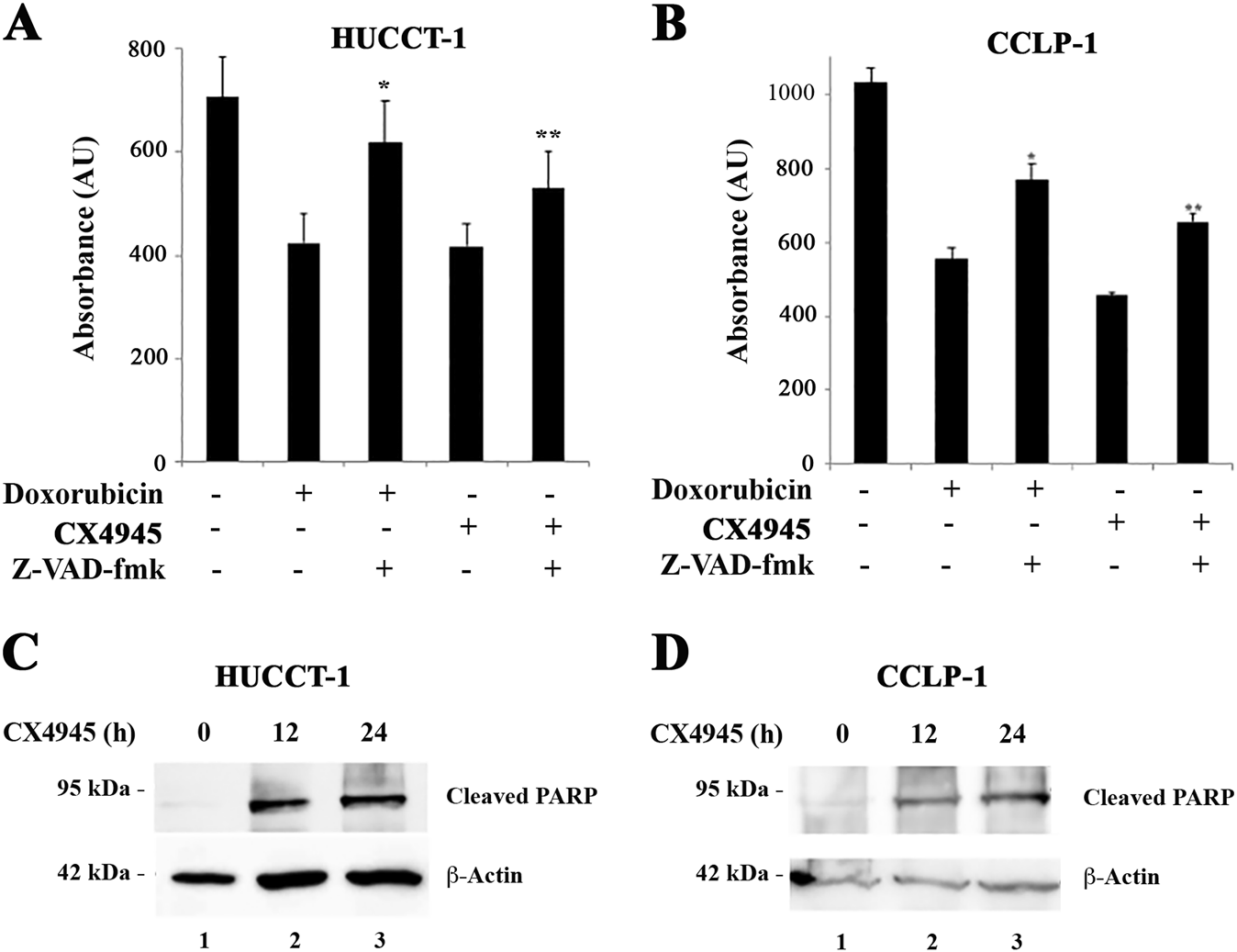
CK2 inhibition induces apoptosis of CCA cells. **a** CCA cells were incubated with doxorubicin (2 μg/ml) or CX4945 (5 μM) for 24 h, in the presence or absence of the pan-caspase inhibitor, z-VAD-fmk (50 μM). MTT assay was performed at the end of the treatment. *p < 0.05 versus Doxorubicin, **p < 0.05 versus CX4945. **b** CCA cells were treated with CX4945 5 µM for the indicated time points. Western blot analysis was performed using the indicated antibodies

We investigated whether the reduction of CCA cell viability in response to CX4945 was due to induction of apoptosis. To this purpose, we pretreated CCA cells with the pan-caspase inhibitor Z-VAD-fmk before adding the CK2 inhibitor and finally measured cell viability. CX4945 treatment caused a reduction of cell viability of approximately 40% in CCA cell lines and this effect was partially rescued by Z-VAD-fmk pretreatment, in a manner similar to the one observed for the well-known pro-apoptotic agent doxorubicin (Fig. 3a, b). This result suggests that CK2 inhibition causes CCA cell death via a caspase-dependent pathway. Consistently, the occurrence of apoptosis in response to CX4945 was confirmed by appearance of the apoptotic PARP fragment in CCA treated cells (Fig. 3c, d).

Because in other studies CX4945 was reported to induce methuosis^24,25^, we checked whether this phenomenon occurred under our experimental conditions. While we confirmed the appearance of large vacuoles in cells treated with 50 µM CX4945, they were detected in less than 5% of the cells at a concentration of 5 μM CX4945 (Supplementary Fig. 2A), which is highly effective in inducing CCA cell death (see Fig. 2).

### CK2 inhibition reduces CCA cell migration and invasion capabilities

**Fig. 4:**
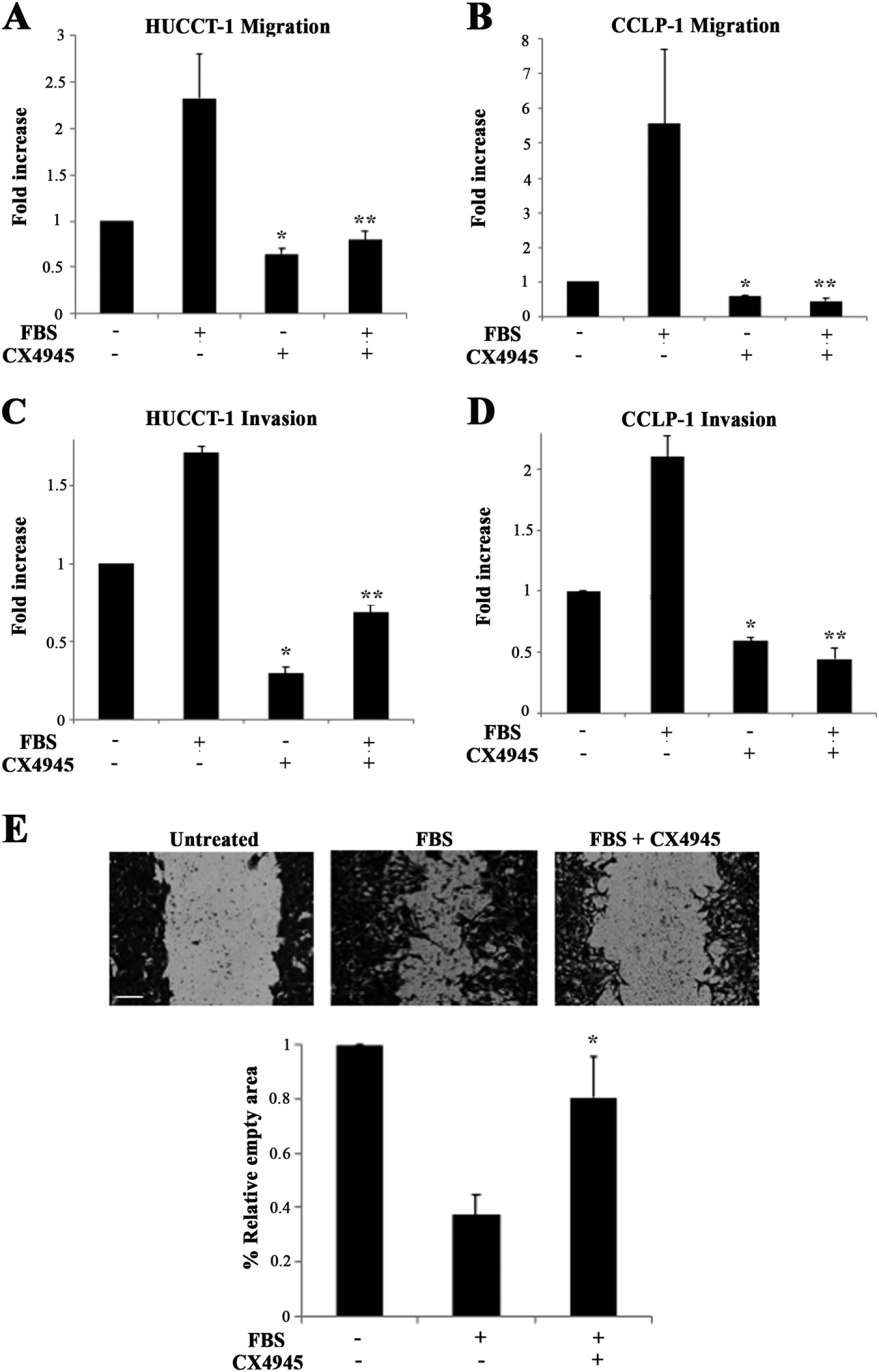
Effects of CK2 inhibition on HUCCT-1 and CCLP-1 motility and invasiveness. Cell migration (**a**, **b**) or invasiveness (**c**, **d**) were evaluated using Boyden chamber assay with 10% FBS as chemotactic stimulus, in the presence or absence of 5 μM CX4945. Before the assay, CCA cells were serum-starved for 24 h. *p < 0.05 vs untreated cells, **p < 0.05 vs. FBS-stimulated cells. **e** Wound-healing assay. HUCCT-1 cells were plated at confluency, scratched with a pipette tip and allowed to heal the wound in 10% FBS-containing medium, in the presence or absence of CX4945 5 µM for 6 h. Images were taken at the end of the experiment (original magnification ×200) and the wound area was quantified using Adobe Photoshop^®^ software. Scale bar corresponds to 200 µm. Data are expressed as the empty area (mean ± SEM) from three independent experiments. *p < 0.05 vs FBS

To further explore the potential role of CK2 in the modulation of the malignant phenotype of CCA cells, we investigated the effects of CK2 inhibition on cell migration and invasion using Boyden chamber assay (Fig. 4a, b). CX4945 treatment reduced the ability to migrate in response to fetal bovine serum, a potent chemotactic stimulus, in both cell lines analyzed. Next, we examined the effects of CX4945 on CCA cell invasion using matrigel-coated inserts to simulate invasion through the extracellular matrix. CK2 blockade markedly and significantly reduced the invasive behavior of CCA cells (Fig. 4c, d). Interestingly CX4945 was able to affect migration and invasion of CCA cells also in the absence of FBS stimulation, indicating a relevant role of CK2 in the highly motile and invasive phenotype of CCA cells even in unstimulated conditions. The latter effect is in keeping with the constitutive activation of CK2 observed in CCA cell lines.

The role of CK2 on CCA cell migration was further assessed with the ‘wound healing’ assay, promoted by FBS in the presence or absence of CX4945. The experiment lasted for 6 h to minimize proliferation-associated effects. As depicted in Fig. 4e, pharmacologic inhibition of CK2 decreased the ability of CCA cells to reduce wound width, confirming the importance of CK2 in the modulation of migratory capabilities of CCA cells.

**Fig. 5:**
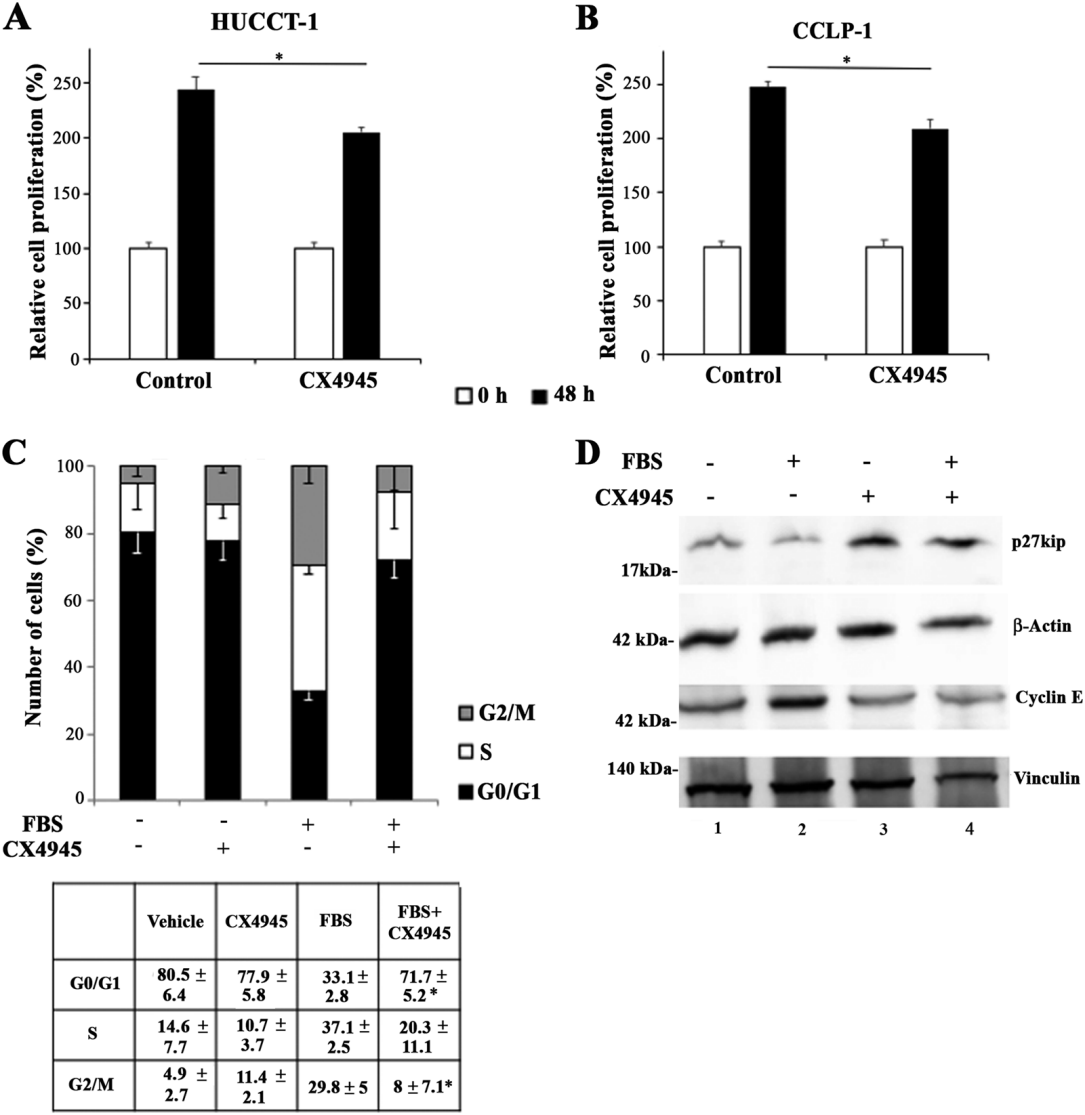
Effects of CK2 inhibition on CCA cell proliferation and cell cycle progression. **a**, **b** CCA cells were serum-starved for 24 h and then stimulated with 10% FBS in the presence or absence of 10 μM CX4945 for 48 h. MTT assay was performed after starvation (0 h) or at the end of the incubation period. *p < 0.05 vs. control. **c** Cultured HUCCT-1 were serum-starved for 24 h and then incubated with 10% FBS in the presence or absence of 10 μM CX4945 for 24 h. Cell cycle phase distribution was determined by flow cytometry. *p < 0.05 vs FBS. **d** Cultured HUCCT-1 were serum-starved for 24 h and then incubated with 10% FBS in the presence or absence of 10 μM CX4945 for 24 h. Western blot analysis was performed using the indicated antibodies

### CK2 modulates proliferation and cell cycle progression in CCA cells

We next examined the functional role of CK2 in the control of proliferation of CCA cells. Upon CK2 inhibition, the time-dependent increase in the number of CCA cells was significantly reduced (Fig. 5a, b). To examine the effect of CK2 inhibition on cell cycle reentry, HUCCT-1 cells were serum starved and then stimulated to reenter the cell cycle by the addition of FBS with or without CX4945. In the presence of serum, about 60% of the cell population entered the S or G_2_/M phase of the cell cycle (Fig. 5c), while in presence of CX4945, the proportion of cells in G_0_/G_1_ phase was comparable to serum-deprived cells. In addition, CK2 inhibition was associated with a decreased expression of cyclin E, a protein essential for G1-to-S phase transition, and a marked increase in the expression of the cyclin dependent kinase inhibitor p27 (Fig. 5d), an important negative modulator of G1 phase checkpoint.

To prove that CK2 inhibition is related to the above described effects on the cell cycle, the experiments were also performed in the presence of the unrelated inhibitor TBB, which resulted in similar effects (Supplementary Fig. 3). In this case, a higher TBB concentration (80 µM) than in cell viability assays was necessary, probably due to the presence of a higher concentration of FBS (10%). Consistently, a marked reduction of CK2 kinase activity was only observed with 80 µM TBB, but not with 20 μM (Supplementary Fig. 4A–B). Importantly, we did not observe any vacuole formation in cells treated with 80 µM TBB, excluding methuosis-like, off-target effects (Supplementary Fig. 4C).

### CK2α CCA knockout cells show reduced proliferation, motility, and cell survival

**Fig. 6:**
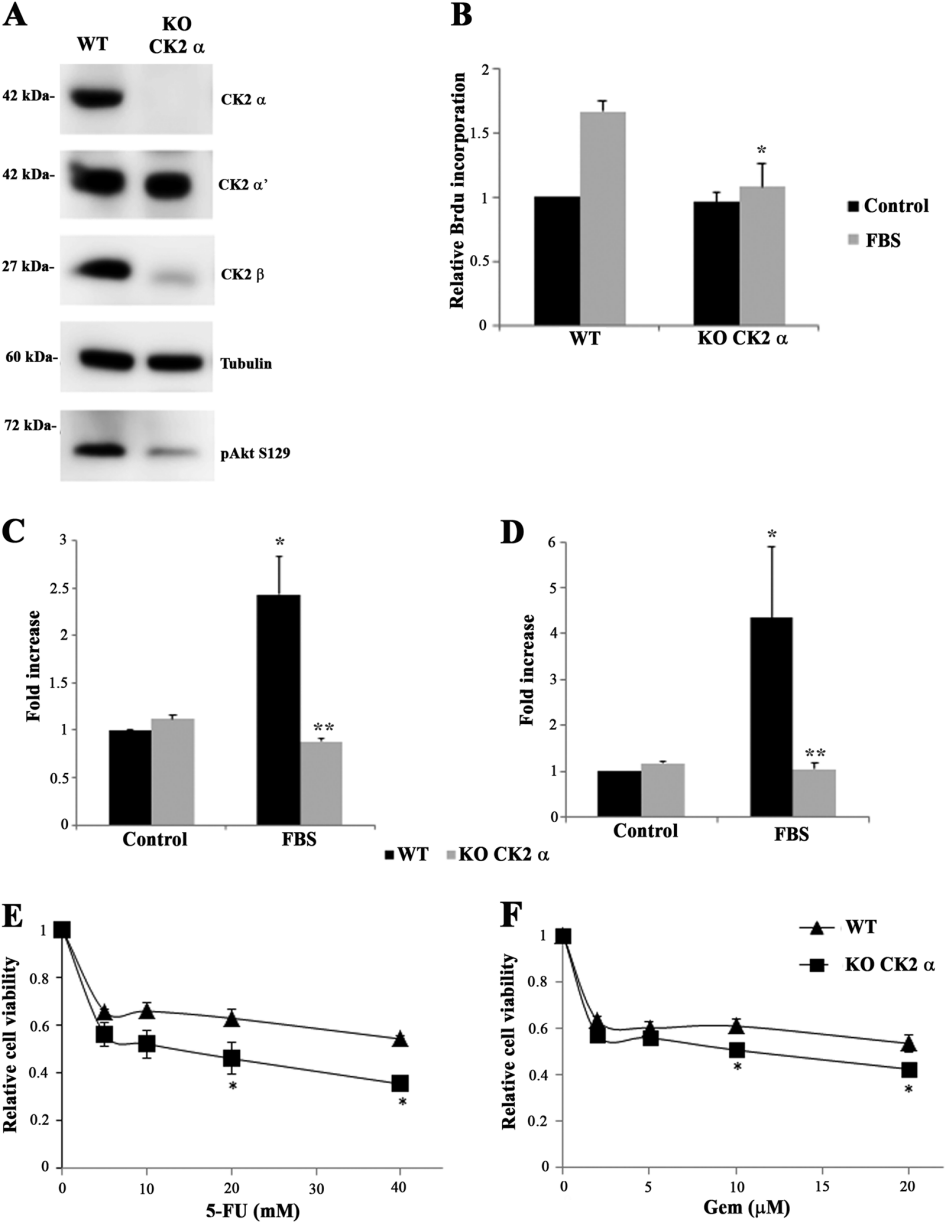
Effects of CK2α knockout on CCA cell biology. **a** Cellular lysates of wild type (WT) and a CK2α knockout clone (KO CK2α) were analyzed by western blot with the indicated antibodies. **b** WT and KO CK2α cells were serum-starved for 24 h and incubated in the presence of 10% FBS for 24 h. Proliferation rate was measured by bromodeoxyuridine (BrdU) incorporation assay. *p < 0.05 vs WT cells treated with FBS. Cell migration (**c**) or invasiveness (**d**) in WT and KO CK2α cells were evaluated using a Boyden chamber assay, with FBS as chemotactic stimulus. Cells were serum-starved for 24 h before the assay. *p < 0.05 vs control cells, **p < 0.05 vs FBS-stimulated cells. **e**, **f** Viability of WT and KO CK2α cells was assessed by MTT after treatment with increasing concentrations of 5-fluorouracil for 48 h or gemcitabine for 72 h. *p < 0.05 vs WT cells treated with the indicated drug at the same concentration

To provide additional evidence that the effects observed by the pharmacologic CK2 blockade were due to CK2 targeting, we knocked out the CK2α catalytic subunit, exploiting the CRISPR/Cas9 genome editing technology. With this technique, we generated a viable clone of HUCCT-1 devoid of CK2α subunit (KO CK2α) (Fig. 6a). Reduced CK2 catalytic activity was confirmed by decreased phosphorylation levels of the endogenous substrate Akt^S129^. We also observed a concomitant strong reduction of the levels of the CK2β regulatory subunit. This finding was not unexpected, since it has been already observed in previous studies and is probably due to a rapid proteolytic degradation of CK2β in the absence of the catalytic subunit^26^.

In CCA cells with this stable non-pharmacological CK2 reduction, we first evaluated the effects on cell proliferation, measuring de novo DNA synthesis. As shown in Fig. 6b, exposure of HUCCT-1 WT cells to FBS for 48 h caused a marked increase of DNA replication, whereas in HUCCT-1 KO CK2α the stimulatory effect of FBS was almost completely abrogated. In addition, KO CK2α cells exhibited a strong reduction in FBS-induced migration and invasion (Fig. 6c, d). Finally, considering the pro-survival and antiapoptotic role of CK2, we checked the sensitivity of KO CK2α cells to 5-fluoracil and gemcitabine, two cytostatic drugs widely used for CCA treatment. We found that deletion of CK2α significantly enhanced the cytotoxicity of both chemotherapeutics, confirming a prominent role of CK2 in mediating survival of CCA cells (Fig. 6e, f).

## Discussion

The identification of effective treatments for CCA requires a better knowledge of the molecular mechanisms involved in growth and spread of cells isolated from this type of cancer. The protein kinase CK2 is not an oncogene per se, but it potentiates signaling pathways driven by oncogenes, thus crucially contributing to the transformed phenotype of malignant neoplasms. Indeed, many cancer cells, at variance with normal ones, are addicted to CK2, and rely on its activity for their survival, proliferation and spreading^10^. In the present study, we provide evidence that CK2 contributes to the malignant phenotype of CCA cells via modulation of key cellular processes. In particular, we demonstrated that CK2 activity is essential for survival, motility and cell cycle progression.

Evidence for the pivotal role of CK2 in the biology of CCA cells was obtained using pharmacologic and genetic approaches in two well-established cellular models (HUCCT-1 and CCLP-1). These cells were shown to overexpress all CK2 subunits and to possess increased CK2 kinase activity compared to non-transformed cholangiocytes. Pharmacologic inhibition of kinase activity with the well-characterized, clinical-grade CK2 inhibitor CX4945 led to a reduction of CCA cell viability, an effect attributable to activation of caspase-dependent apoptosis. In addition, CK2 activity was implicated in the control of local spread and metastatic potential of CCA cells, as demonstrated by the reduction of the migratory and invasive capacity of HUCCT-1 and CCLP-1 exposed to CX4945. These results expand previous observations demonstrating that CX4945 and the TGF-β inhibitor LY2157299 have cooperative and complementary effects on CCA survival and migration^20^. CK2 inhibition reduced cell proliferation and blocked G1 to S phase progression. Growth arrest was associated with increased expression of p27 and reduction of cyclin E, two important regulators of the G1-S transition, the expression of which is characterized by an inverse relationship, in agreement with our data. Modulation of p27 by CK2 has been reported in other types of cancer cells^27^, whereas the connection between CK2 and Cyclin E is a novel finding which defines an additional regulatory role of CK2 during cell cycle progression. Given the relevance of this pathway in cancer biology, further studies are warranted to clarify the molecular mechanisms linking CK2 with the CDK2/cyclin E pathway.

Although CX4945 is considered quite selective^28^, other CK2-independent functions have been ascribed to CX4945, such as the suppression of phosphorylation of serine/arginine (S/R) rich proteins with effects on alternative splicing of a wide range of genes^23^. Moreover, CX4945 was found to induce methuosis, a non-apoptotic form of cell death accompanied by the formation of large fluid-filled vacuoles, reported in both colon cancer^25^ and CCA cells^24^. We checked the occurrence of methuosis in CX4945-treated cells, but this was virtually not detected at drug concentrations (5–10 µM) used in this study. Since our treatment at 5 µM resulted in around 70% cell death, the effects of CX4945 on CCA observed in this study are unlikely to be due to methuosis. On the other hand, the possibility that other forms of cell death contribute to the effects of CX4945 cannot be completely ruled out, as indicated by fact that a pan-caspase inhibitor did not completely revert the actions of this drug on cell viability.

To provide additional evidence that the observed effects on CCA cells are due to CK2 inhibition, we confirmed our major findings using TBB, a CK2 inhibitor unrelated to CX4945^22^. Indeed, reduced cell viability and block of cell cycle in the G0/G1 phase were also observed in cells exposed to TBB. Moreover, we exploited the CRISPR/Cas9 genome editing technology to generate viable CCA cells devoid of the α catalytic subunit of CK2. Considering that chemical targeting of CK2 by CX4945 is expected to block both the α and α‘ isoform^29,30^, we tried to obtain a double α/α′ knock-out in HUCCT-1 cells, but all attempts were unsuccessful, since cells were not viable. This negative result is *per se* interesting, since it confirms the ‘addiction’ of CCA cells to CK2 for their survival. Indeed, only non-transformed cells completely devoid of CK2 catalytic activity have been successfully generated so far^26^. Nevertheless, despite using cells where only the α subunit had been knocked down, a strong reduction of the malignant features of CCA cells was observed. Specifically, proliferation, migration, invasion, and survival when exposed to cytostatic drugs were markedly and significantly reduced in cells depleted of the CK2 α subunit. Thus, complete abrogation of CK2 activity does not appear to be necessary to negatively modulate the aggressive phenotype of CCA cells. An alternative hypothesis is that CK2 has isoform-specific functions for the α subunit, not shared by α′, in determining the aggressive properties of CCA. Although the α and α′ CK2 subunits are highly conserved in sequence and usually considered overlapping in function, they have also been reported to have specific roles^31^. Future work will be necessary to confirm or exclude this possibility, in the context of CCA biology.

The results obtained in cultured CCA cells are markedly strengthened by the analysis of transcriptome datasets from surgically resected CCA specimens, which showed elevated expression of CK2 catalytic and regulatory subunits in the tumor in comparison to matched surrounding non-tumor tissue. These data are in agreement with a previous study that reported overexpression of the CK2α and CK2β genes in several types of lethal cancers including hepatocellular carcinoma^32^, and with data proposing a correlation between overexpression of CK2β and CCA progression^33^.

In summary, our data strongly indicate that CK2 contributes to the aggressive phenotype of CCA cells through modulation of cell survival, cell cycle and cell motility, and indicate that CCA cells with reduced CK2 activity are more sensitive to conventional antitumor drugs. Of note, most data were obtained using a pharmacologic inhibitor that has been qualified for clinical trials. While our investigation was performed at a molecular and cellular level, another recent study has demonstrated that CX4945 is effective in reducing the growth of CCA cells in an in vivo xenograft model in mice^19^, synergizing with conventional drugs. Based on the results from our group and from other scientists, CK2 targeting merits future evaluation as an additional approach to the treatment of CCA, in combination therapies.

## Materials and methods

### Reagents

CK2α (C-terminal) antibodies were raised in rabbit^34^, CK2α (N-terminal) (Cat N.: MCA3031Z) antibody was from Biorad Laboratories (Hercules, CA, USA), CK2β (Cat N.: ab76025) and p-Akt1 S129 (Cat N.: ab133458) antibodies were from Abcam (Cambridge, UK). Cleaved PARP (Cat N.: #9541) and p27Kip (Cat N.: #2552) antibodies were from Cell Signaling Technology (Danvers, MA, USA), Vinculin (Cat N.: V9131), α-Tubulin (Cat N.: T5168**)** and Actin (Cat N.: A5441) were from Sigma-Aldrich (St Louis MO, USA). Akt1 (Cat N.: sc-1618) and Cyclin E (Cat N.: sc-481) antibodies were from Santa Cruz Biotechnology (Santa Cruz, CA, USA). Crispr/Cas9 all-in-one plasmids were purchased from ATUM^SM^.CX4945 was from Glixx Laboratories (Hopkinton, MA, USA). TBB was kindly provided by Dr. Z. Kazimierczuk, Warsaw, Poland; Caspase inhibitor Z-VAD-FMK was from Santa Cruz Biotechnology (Santa Cruz, CA, USA). Doxorubicin, 5-Fluoruracil (5-FU) and Gemcitabine were from Sigma-Aldrich (St Louis MO, USA).

### CCA patient database

The GSE26566 series matrix containing expression values from Illumina humanRef-8 v2.0 expression beadchip arrays [transcript (gene) version] of 104 CCA patients was downloaded from GEO^35^. Differences in gene expression of specific genes of 104 tumor tissues (T) versus 60 matched surrounding liver (SL) were evaluated. All samples were obtained with approval by the institutional review board of the National Institutes of Health and collaborating institutions on the condition that patients were anonymized.

### Cell culture

CCA cell lines (HUCCT-1, CCLP-1) used in this study were kindly provided by Dr. A.J. Demetris, University of Pittsburgh and Dr. G. Alpini, Texas A&M Health Science Center, USA. Cells were cultured according to conditions described elsewhere^36^. The primary human intrahepatic cholangiocyte cell line HiBEC (indicated as “cholangiocytes”) was from ScienCell. Experiments were performed using cells between passages 2 and 8. All cell lines were incubated at 37 °C in a humidified chamber supplemented with 5% CO_2_. All cells used were negative for mycoplasma (Mycoplasma detection kit, Roche, Germany).

### Measurement of cell viability

Cell viability was detected by means of 3-(4,5-dimethylthiazol-2-yl)-3,5-diphenyltetrazolium bromide (MTT) reagent, a tetrazolium salt that is metabolized by mitochondrial dehydrogenases and produces a purple precipitate in viable cells. Cell suspension (1 × 10^4^ to 2.5 × 10^4^ cells in 100 μl) was incubated in each well of a 96-well plate under different conditions, in the culture medium but with 1% (instead of 10%) FBS, where not differently indicated. Then, 2 h before the end of the incubations, 10 μl of MTT solution (5 mg/ml in PBS) was added to each well. Incubations were stopped by addition of 20 μl of lysis solution at pH 4.7, consisting of 20% (w/v) SDS, 50% (v/v) *N*,*N* dimethylformamide, 2%(v/v) acetic acid and 25 Mm HCl. Plates were read for absorbance at 570 nm, in a Multiskan FC plate reader (Thermo Scientific, Waltham, MA, USA).

### Analysis of cell cycle

Eighty thousands cells/well were seeded in multi-wells dishes and exposed to the appropriate conditions. After medium removal, 400 μl of solution containing 50 μg/ml propidium iodide, 0.1% w/v trisodium citrate, 0.1% NP40 was added. Samples were then incubated for 30 min at 4 °C in the dark and nuclei analyzed with a FACSCanto flow cytometer (Becton Dickinson, Franklin Lakes, NJ, USA).

### BrdU incorporation assay

BrdU incorporation assay was performed using the following colorimetric immunoassay: Cell Proliferation ELISA, BrdU (colorimetric) Kit (Roche Applied Science, Indianapolis, IN) based on the measurement of BrdU incorporation. In total, 15,000 cells/well were seeded in 96-well plates, incubated in complete medium for 24 h and serum-starved for additional 24 h. The assay was performed following the manufacturer’s protocol.

### Wound healing assay

HUCCT-1 cells (20,000 cells/well) were seeded in six-well plates and allowed to form cell monolayer. Confluent CCA cells were serum starved for 16 h. After serum starvation, a sterile 10 µl plastic pipette tip was used to create a line-shaped wound across the cell monolayer, and the detached cells were removed by rinsing with phosphate-buffered saline (PBS). Next, cells were stimulated with 10 % FBS in presence or absence of CX4945 5 µM for 6 h. Images were taken at the end of the experiment (original magnification ×200) and the wound area was quantified using Adobe Photoshop® software.

### CRISPR/Cas9-mediated genome editing

CK2α knockout clone was generated by Crisp/Cas9-mediated genome editing as described in Borgo et al.^26^. All-in-one plasmids expressing Cas9-DasherGFP and the sgRNA guide (pD1301-AD: CMV-Cas9-2A-GFP, Cas9-ElecD) to target the specific CK2 subunit was used. The sgRNA guide sequences are 5′- CCTGGATTATTGTCACAGCA-3′ (CK2α). Briefly, HUCCT-1 cells were cultured in six-well dishes to 70–80% confluence. Cells were co-transfected with 1 μg of sgRNA plasmid and Lipofectamine 3000 according to manufacturing instructions. Forty-eight hours post-transfection, cells were pelleted in PBS and sorted in 96-well plates using fluorescence-activated cell sorting (FACS) with a FACSAria II cell sorter (BD BioSciences). Single cells were expanded to obtain individual clones. Individual clones were lysed and quantified as described above. The absence of CK2α was verified by western blotting.

### CK2 activity assay in cell lysates

Proteins from cell lysates (1–2 μg) were incubated for 10 min at 30 °C in 25 μl of a phosphorylation medium containing 50 mM Tris–HCl (pH 7.5), 100 mM NaCl, 10 mM MgCl_2_, 400 μM synthetic peptide-substrate RRRADDSDDDDD and 50 μM [γ-^33^P] ATP (1000 cpm/pmol). Assays were stopped by absorption onto phosphocellulose filters. Filters were washed four times in 75 mM phosphoric acid and analyzed by a Scintillation Counter (PerkinElmer).

### RNA isolation and real time quantitative PCR

Total RNA from cells was isolated using the RNeasy kit (QIAGEN Sciences, MD) according to the manufacturer’s recommendations. The RNA concentration and quality were measured using an optical Nanodrop ND1000 spectrophotometer (Thermo Scientific). Total RNA (1500 ng) was reverse transcribed into cDNA using iScript Adv cDNA kit (Biorad) according to the manufacturer’s instructions. Changes in the mRNA expression level of target genes were detected using SosAdvanced Universal SYBR-Green SuperMix and the Corbett Rotorgene 6600 analyzer (Corbett Life Science, Sydney, Australia). Relative gene expression was calculated as 2^−ΔCt^ (ΔCt = Ct of the target gene minus Ct of GAPDH).

We used the following pairs of primers to analyze the gene expression:

CK2α forward 5′-TGTCCGAGTTGCTTCCCGATACTT-3′

CK2α reverse 5′-TTGCCAGCATACAACCCAAACTCC-3′

CK2α’ forward 5′-AGCCCACCACCGTATATCAAACCT-3′

CK2α’ reverse 5′-ATGCTTTCTGGGTCGGGAAGAAGT-3′

CK2β forward 5′-TTGGACCTGGAGCCTGATGAAGAA-3′

CK2β reverse 5′-TAGCGGGCGTGGATCAATCCATAA-3′.

The real-time PCR results are normalized against the control GAPDH housekeeping gene using the primers:

forward 5′-CAACGACCACTTTGTCAAGC-3′

reverse 5′-TCTTCCTCTTGTGCTCTTGC -3′

### Western Blot analysis

Procedures for preparation of cell lysates, sodium dodecyl sulfate polyacrylamide gel electrophoresis analysis and Western blotting have been described elsewhere^37^. Quantification of the signal was obtained by chemiluminescence detection on a Image Quant Las4000 (GE Healthcare Life Sciences) and subsequent analysis with the ImageJ or Adobe Photoshop software.

### Measurement of cell migration and invasion

Migration of HUCCT-1 or CCLP-1 cells was assayed using modified Boyden chambers, Briefly, 20,000 cells were seeded in wells equipped with 8-μm pore filters (Millipore Corp, MA, USA) and coated with rat tail collagen (20 μg/ml) (Collaborative Biomedical Products, Bedford, USA) or Matrigel (150 μg/ml) (BD Biosciences, MA, USA), to be used for chemotaxis and chemoinvasion assays, respectively. After the time of incubation (16 h), the migrated or invaded cells were fixed, stained with Giemsa, mounted counted at ×40 magnification. Data are the average of cell counts obtained in 12 randomly chosen high-power fields (HPF).

### Statistical analysis

Autoluminograms and immunofluorescence images are representative of at least three experiments with comparable results. All barograms show the combined results of three independent experiments, shown as mean ± SEM. Unless specified otherwise, paired two-tailed Student’s *t* test was applied to determine statistical significance, for normally distributed samples. *P* values lower than 0.05 were considered significant.

## Supplementary information

Supplementary Figure legends

Supplementary Figure 1

Supplementary Figure 2

Supplementary Figure 3

Supplementary Figure 4
