# Supplementary material for: The protein kinase CK2 contributes to the malignant phenotype of cholangiocarcinoma cells
Source: Oncogenesis. 2019 Oct 22;8(11):61. doi: 10.1038/s41389-019-0171-x (PMC6805921; doi:10.1038/s41389-019-0171-x)
Supplement: Supplementary file 1 — Supplementary Figure legends [file 41389_2019_171_MOESM1_ESM.docx]

**SUPPLEMENTARY FIGURE LEGENDS**

**Supplementary Figure 1: Effects of the CK2 inhibitor, TBB, on cell viability in CCA cells.**

CCA cells were treated with the indicated concentrations of TBB for 48 h. MTT assay was performed at the end of the treatment. *p<0.05 vs. control.

**Supplementary Figure 2: Effect of CK2 inhibitor CX4945 on vacuole formation in CCA cells.** CCA cells (HUCCT-1 in panel A, CCLP-1 in panel B) were treated with the indicated concentrations of CX4945 for 24 h. At the end of experiment cells were stained with Giemsa. Pictures are representative of the results of three independent experiments.

**Supplementary Figure 3: Effects of CK2 inhibitor TBB on CCA cell cycle progression.** Cultured HUCCT-1 were serum-starved for 24 hours and then incubated with 10 % FBS in presence or absence of 80 μM TBB for 24 h. Cell cycle phase distribution was determined by flow cytometry. *p<0.05 vs. FBS.

**Supplementary Figure 4: Effects of CK2 inhibitor TBB on CCA cell CK2 activity and vacuole formation.**

(A) Cultured HUCCT-1 cells were treated with vehicle (Control) or the indicated concentrations of TBB for 24h. Activity of CK2 in cell lysates was measured towards the CK2-specific peptide substrate. *p<0.05 vs. vehicle. (B) Cultured HUCCT-1 cells were treated with vehicle or the indicated concentrations of TBB for 24 h. Twenty-five µg of proteins from total cell lysate were analyzed by western blot with the indicated antibodies. (C) Cultured HUCCT-1 were serum-starved for 24 hours and then incubated with 10 % FBS in presence or absence of 80 μM TBB for 24 h. At the end of experiment, cells were stained with Giemsa. Pictures are representative of the results of three independent experiments.
