# Supplementary figures and images for: The protein kinase CK2 contributes to the malignant phenotype of cholangiocarcinoma cells
Source: Oncogenesis. 2019 Oct 22;8(11):61. doi: 10.1038/s41389-019-0171-x (PMC6805921; doi:10.1038/s41389-019-0171-x)

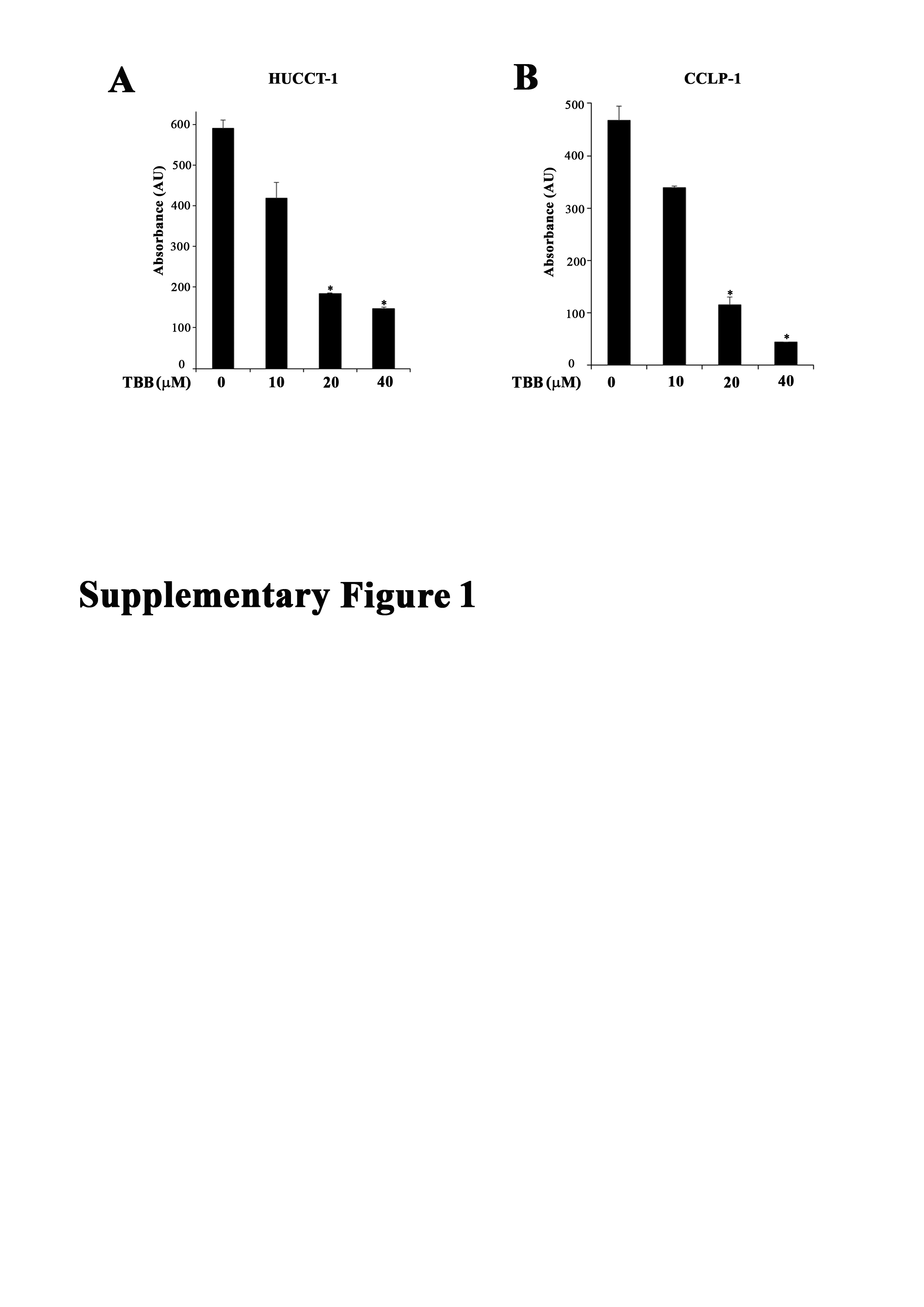

Supplement: Supplementary file 2 — Supplementary Figure 1 [file 41389_2019_171_MOESM2_ESM.tif]

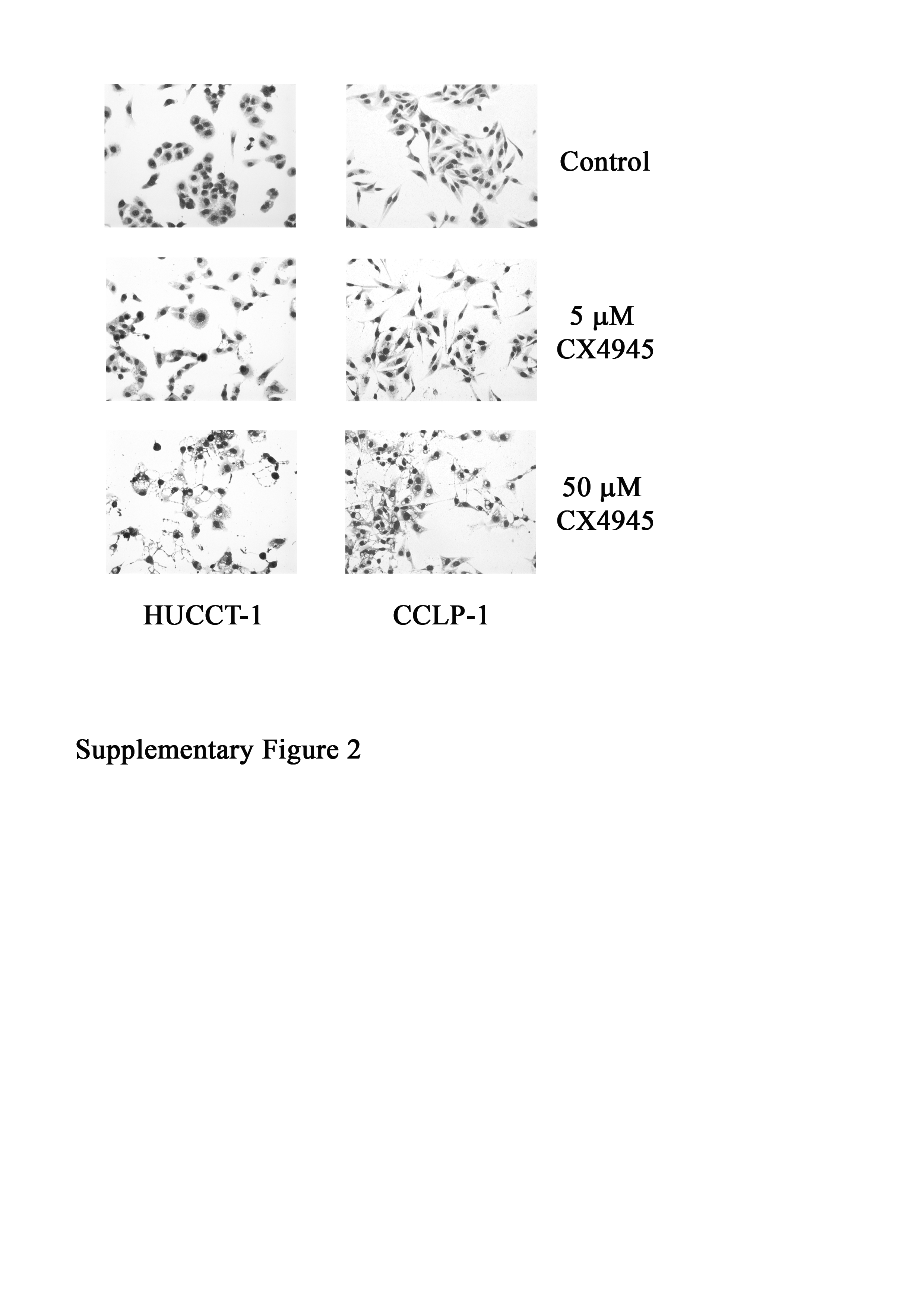

Supplement: Supplementary file 3 — Supplementary Figure 2 [file 41389_2019_171_MOESM3_ESM.tif]

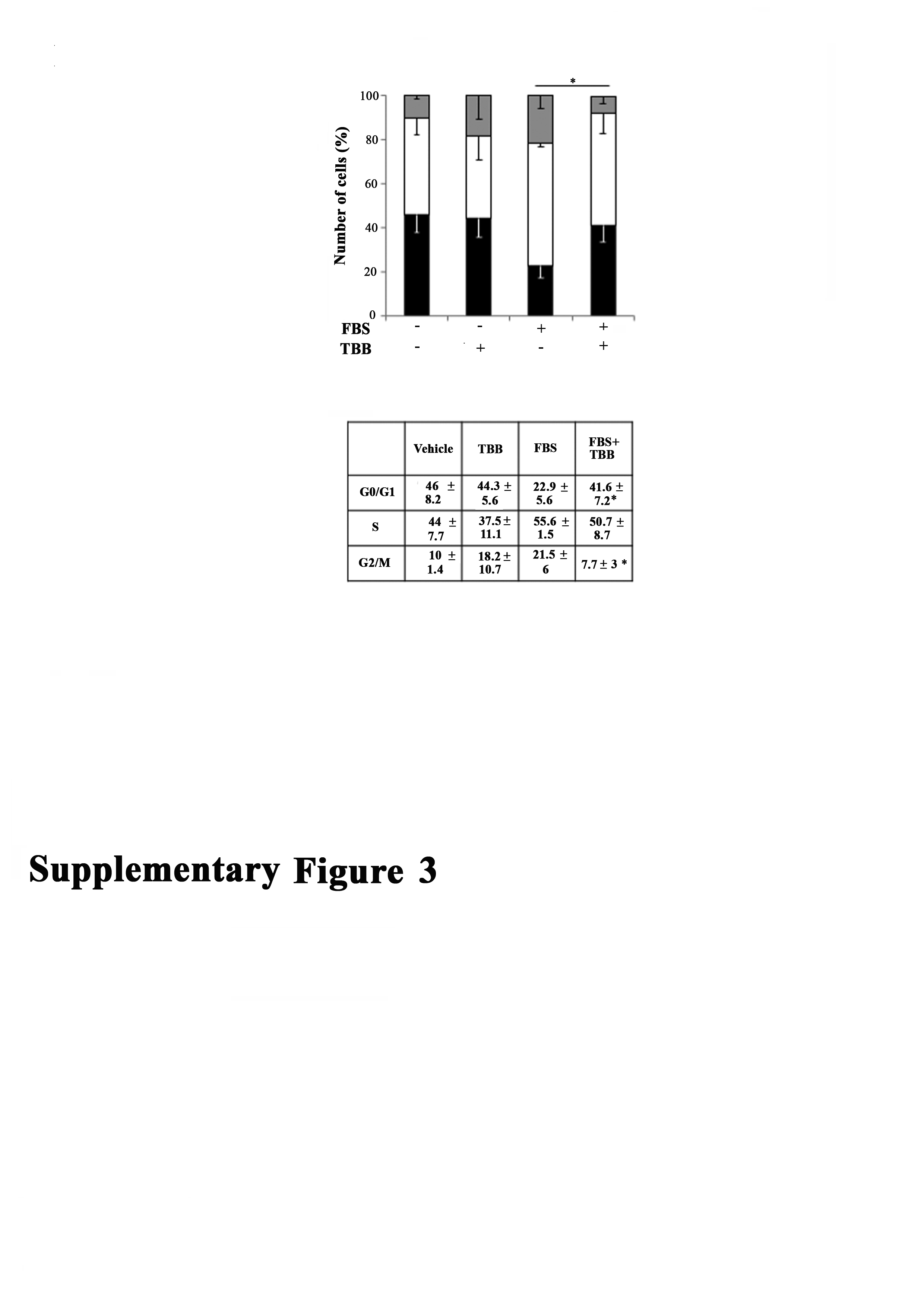

Supplement: Supplementary file 4 — Supplementary Figure 3 [file 41389_2019_171_MOESM4_ESM.tif]

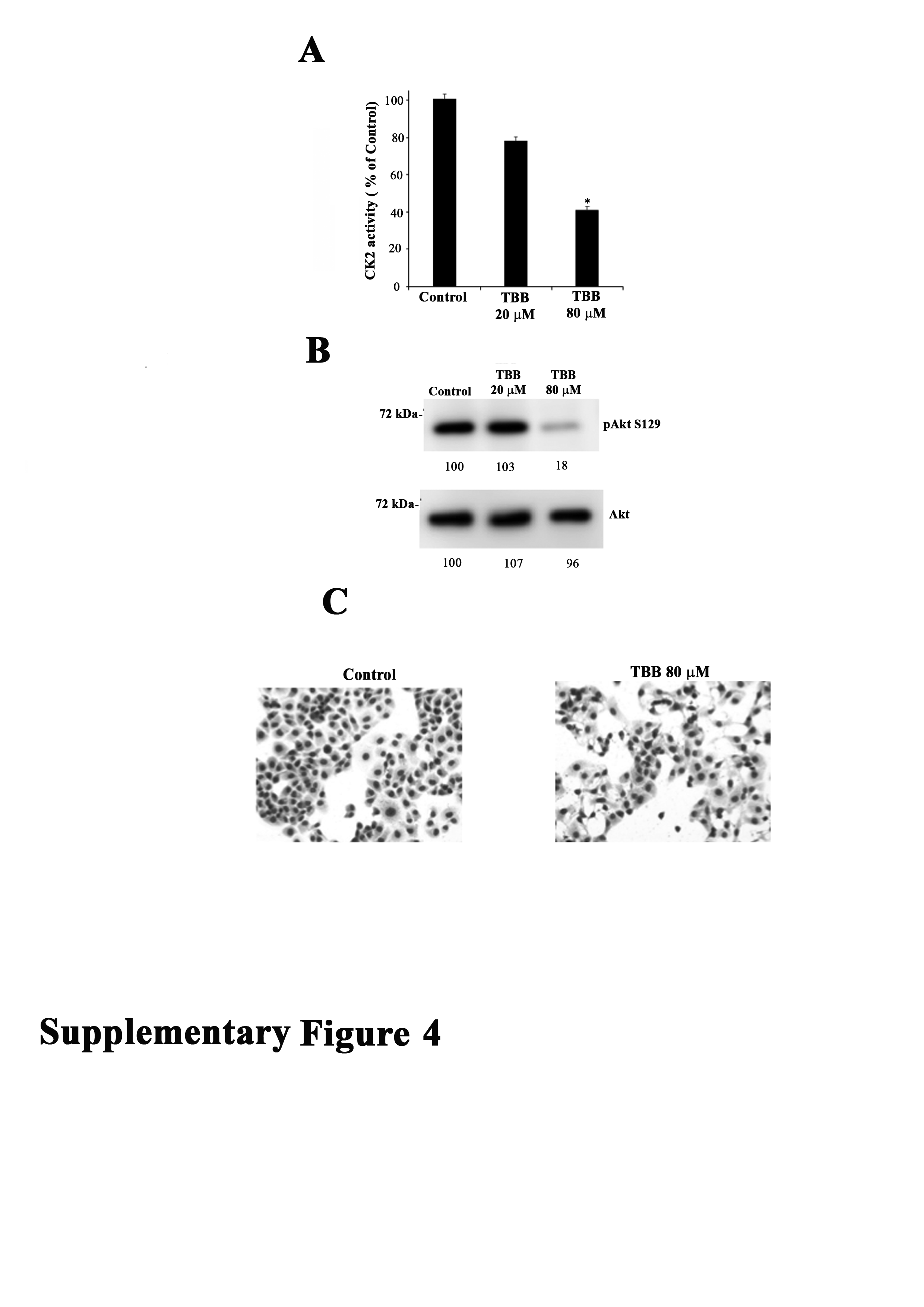

Supplement: Supplementary file 5 — Supplementary Figure 4 [file 41389_2019_171_MOESM5_ESM.tif]
